# Proceedings of the Fifth Biennial Conference of the Society for Implementation Research Collaboration (SIRC) 2019: where the rubber meets the road: the intersection of research, policy, and practice - part 1

**DOI:** 10.1186/s13012-020-01034-7

**Published:** 2020-09-30

**Authors:** Sara J. Landes, Suzanne E. U. Kerns, Meagan R. Pilar, Callie Walsh-Bailey, Stephanie H. Yu, Y. Vivian Byeon, Margaret E. Crane, Madeline Larson, Heather L. Bullock, Ana A. Baumann, Katherine Anne Comtois, Doyanne Darnell, Shannon Dorsey, Phil Fizur, Cara C. Lewis, Joanna C. Moullin, Andria Pierson, Byron J. Powell, Cameo F. Stanick, Shannon Wiltsey Stirman, Robert P. Franks

**Affiliations:** 1 Department of Psychiatry, University of Arkansas for Medical Sciences, Little Rock, AR USA; 2 Behavioral Health QUERI, Central Arkansas Veterans Healthcare System, North Little Rock, AR USA; 3 South Central Mental Illness Research Education and Clinical Center, Central Arkansas Veterans Healthcare System, North Little Rock, AR USA; 4 Graduate School of Social Work, University of Denver, Denver, CO USA; 5 Brown School, Washington University in St. Louis, St. Louis, MO USA; 6 Department of Psychology, University of California, Los Angeles, Los Angeles, CA USA; 7 Department of Psychology, Temple University, Philadelphia, PA USA; 8 Department of Educational Psychology, University of Minnesota, Twin Cities, Minneapolis, MN USA; 9 McMaster Health Forum, Department of Health Research Methods, Evidence and Impact, McMaster University, Hamilton, ON Canada; 10 Waypoint Centre for Mental Health Care, Penetanguishene, ON Canada; 11 Department of Psychiatry and Behavioral Sciences, University of Washington, Seattle, WA USA; 12 Department of Psychology, University of Washington, Seattle, WA USA; 13 Division of Behavioral Medicine, Cooper University Hospital, Camden, NJ USA; 14 Cooper Medical School, Rowan University, Camden, NJ USA; 15 MacColl Center for Healthcare Innovation, Kaiser Permanente Washington Health Research Institute, Seattle, WA USA; 16 Faculty of Health Sciences, Curtin University, Bentley, WA Australia; 17 Clinical Practice, Training, and Research and Evaluation Department, Hathaway-Sycamores Child and Family Services, Pasadena, CA USA; 18 National Center for PTSD, VA Palo Alto Health Care System, Menlo Park, CA USA; 19 Department of Psychiatry and Behavioral Sciences, Stanford University, Stanford, CA USA; 20 Judge Baker Children’s Center/Department of Psychiatry, Harvard Medical School, Boston, MA USA

## Introduction

The Society for Implementation Research Collaboration (SIRC) evolved as a society following a National Institute of Mental Health (NIMH)-funded conference grant (NIMH 1R13MH086159-01A1, PI Comtois) and has continued to develop as an international society [1]. At the 5th biennial conference held in in Seattle, WA, USA, on September 12–14, 2019, we announced that we had incorporated SIRC as an entity and obtained non-profit status with generous assistance from the Business Innovations Clinic at the University of Arkansas Little Rock, Bowen School of Law. SIRC’s goal is to improve the implementation of effective practices in behavioral health, health, and social care, notably through collaboration among communities, researchers, purveyors of evidence-based practices, practitioners, and policy makers. To include and support a variety of member types, SIRC created Networks of Expertise (NoE). These networks include the Student, New Investigator, Established Investigator, Practitioner, and Mechanisms NoE. Each NoE focuses on activities relevant to their members, such as pairing Students with New or New with Established Investigator mentors, hosting office hours to share expertise, and developing conference content relevant to their interests. Conference activities related to the NoEs are highlighted throughout this summary.

The 2019 conference, entitled “Where the Rubber Meets the Road: The Intersection of Research, Policy, and Practice,” brought together 432 attendees from across the globe with interests in implementation research, policy, and practice within and outside of academia. The conference theme was inspired by a discussion during a breakout session at a prior SIRC conference. One of the practitioner attendees suggested that SIRC further emphasize how researchers take what they learn from implementation science and better collaborate with practitioners to apply the concepts on the ground. This participant referred to this as “where the rubber meets the road.” The robust conversation that followed seeded our 2019 conference theme. This theme was operationalized within the 2019 conference through intentional programmatic structuring that provided ample opportunities for dialogue. For example, time was allocated in each conference breakout session and plenary to facilitate dialogue and cross-collaboration between researchers, practitioners, policy makers, and students. Lunch sessions and social events provided more possibilities for casual discussion and exchanges of ideas. The main conference reception intentionally followed ignite presentations (described below) to enable timely interactions between speakers and conference attendees. We also scheduled a longer poster session and grouped posters thematically to enable interest groups to form. Plenary presentations were selected that highlighted the conference theme and the conference committee included the conference theme as one of the review criteria to prioritize selection of symposium and papers for the conference that were aligned.

## Preconference invited implementation development workshops

SIRC hosted four implementation development workshops (IDWs) for 54 invited attendees who were also members of a SIRC NoE. IDWs are designed for presenters to get feedback on implementation research and/or projects that are currently in development (as opposed to completed work presented during the main conference). As described in our methodology paper [2], an IDW includes approximately twelve attendees, four to five presenters, a facilitator, and note takers. Each presenter is given 45 min for their project with 10–20 min focused on their presentation. Presenters provide a 1-page description of their project and identify questions for feedback; no technology is allowed or used. A facilitator manages the time and coordinates discussion. Note takers record feedback and discussion, which allows for presenters to participate more fully. To increase the degree to which projects evaluated mechanisms (i.e., how implementation strategies work) [3], IDWs included individuals from the Mechanisms NoE (described below).

## Preconference open workshops

Five workshops were available to all conference attendees. Workshop topics aligned with SIRC’s goals of advancing implementation research and practice. The first workshop focused on creating implementation laboratories [4] to efficiently advance implementation science and practice (presented by Jeremy Grimshaw and Noah Ivers). The second workshop was led by SIRC Practitioner NoE members and sponsored by the Annie E. Casey Foundation. This workshop facilitated discussion about developing system-wide infrastructure to support evidence-based practice implementation and sustainability and kicked-off an ongoing project to generate guidance on this topic (presented by Alexia Jaouich, Purnima Sundar, Heather Bullock, Carrie Comeau, Amberlee Venti, Blair Brooke-Weiss, and Gery Shelafoe). The third workshop focused on education and training in implementation science to advance research and practice and was sponsored by the Centre for Evidence and Implementation (presented by Robyn Mildon and Joanne Yoong). The fourth workshop taught attendees how to communicate beyond the academy, to the public, press, and policy makers (presented by Beth Prusaczyk). The fifth workshop was added to address past participant requests and was an introduction to implementation science designed to prepare conference attendees with little previous exposure to implementation science (presented by Byron Powell, Rinad Beidas, and Shannon Wiltsey Stirman).

## Main conference summary

The 2019 meeting featured four plenary sessions, opening and closing each of the 2 days. Day 1 kicked off with a plenary presentation from Arthur C. Evans, Jr., the CEO of the American Psychological Association and former Commissioner of Philadelphia’s Department of Behavioral Health and Intellectual disAbility Service, titled “Where the Rubber Meets the Road: Reframing Implementation to Advance Science and Practice.” This presentation tackled multiple approaches to increase the relevance of implementation science to everyday practice. For example, Dr. Evans discussed the importance of integrating change initiatives that involve evidence-based practices into providers' current therapeutic approaches and that cultural considerations can be integrated within implementation practice.

Day 1 closed with a series of ignite format plenary presentations. Ignite talks are a series of short presentations; each speaker has 5 min to present and are allowed 20 presentation slides that automatically advance every 15 s. The goal is to “ignite” interest and, as such, the ignite plenary session was held prior to the conference reception to facilitate conversation among attendees. The ignite plenary topics included implementing service cascade models with fidelity (presented by Alicia Bunger), a pragmatic method for costing implementation strategies (Zuleyha Cidav), communication between implementation researchers and social entrepreneurs (Cole Hooley), implementation efforts for system level change in child welfare (Melissa Bernstein), a tailored approach to implementing measurement-based care (Byron Powell), intervention mapping to adapt evidence-based interventions (Maria Fernández), co-creation of change in policy and practice (Gregory Aarons), and developing a strategic implementation research plan (Sara Landes).

Day 2 of the conference began with a third plenary session that included Lilian Nandutu Aluka, Bernard Wafula Nambafu Nabalia, Sheila Wambui Nderitu, Omariba Anne Nyaboke, and Daisy Anyango Okoth of Ace Africa Kenya and Shannon Dorsey of University of Washington. This plenary was titled “Overcoming Implementation Challenges in Low-Resource Communities: Methods and Solutions from Western Kenya.” First, the science informing the implementation approach was described. Then, the experienced lay counselors from Ace Africa described the community collaboration methods used to identify implementation practices and policies that supported successful implementation of a mental health intervention for youth in both the education and health sectors. They described how this work informed the development and delivery of an implementation coaching intervention designed to fit to low-resource context and allow tailoring to individual sites. Day 2 ended with a final plenary session with leaders in the field sharing their perspectives on the intersection of implementation research, policy, and practice, as well as their views on the way forward. Entitled “Perspectives on the Intersection of Implementation Research, Policy and Practice: Offering a Path Forward,” this plenary panel included Melanie Barwick, Jeremy Grimshaw, Lisa Saldana, and Bryan Weiner and was moderated by Robyn Mildon.

The 2019 SIRC conference included 25 breakout sessions addressing multiple content areas. Each breakout session focused on different aspects of implementation science, including outer contexts, collaboration, leveraging collaborative care, mechanisms of implementation, leadership, role of intermediary organizations, behavioral economics and participatory design, the role of implementation science in achieving health equity, economic methods, adaptations, and measurement. Some sessions were context specific, such as focusing on implementation within health care, schools, communities, public health systems, and within policy contexts.

A poster session was scheduled for 90 min to provide ample opportunity for discussion and networking. Poster presenters were encouraged to use the “better poster” design to enhance conversations [5]. The better poster design highlights a main finding in large font and is intended to make it easier for conference attendees to see key ideas. Posters were grouped by themes. Particular attention was paid to grouping posters by implementation context (e.g., community mental health, primary care, school settings) or area of focus (e.g., cancer, chronic illness, food/nutrition, substance use). Additionally, several poster groupings focused on training and workforce development, sustainability, technology, and international implementation efforts.

To facilitate interaction among attendees, SIRC hosted multiple social events including three lunch events during the conference. One lunch event allowed attendees to interact with the *Implementation Science Communications* journal co-editor-in-chief, Anne Sales. After a brief introduction to the goals and mission of the recently launched journal, participants were encouraged to ask questions and engage in a dialogue about preparing manuscripts. Another lunch event was hosted by the Practitioner NoE, which includes provider, intermediaries, and policy/funder sub-networks. This lunch event allowed networking among practitioners and discussion of what each sub-network would like to accomplish in the coming years. The third lunch event was focused on SIRC student development and networking. The goal of this lunch event was to explore the numerous career possibilities and trajectories in the field of implementation. Attendees were invited to lunch with experts working at various levels of the implementation process, including administrators, intermediaries, policy makers, researchers, and service providers. SIRC hosted evening social events as well, including dinners and a boat cruise.

## Mechanisms Network of Expertise

SIRC’s 4th biennial conference, held in Seattle in 2017, was centered on the theme: Implementation Mechanisms: What Makes Implementation Work and Why [6]? Inspired by the rich dialogue of this previous conference, and the observation that few scholars are investigating implementation mechanisms, SIRC partnered with independent investigators and secured funding from the Agency for Healthcare Research and Quality to support the conference series in developing and disseminating an implementation mechanisms research agenda. Led by principal investigator Cara Lewis, SIRC assembled a Mechanisms Network of Expertise (MNoE; for more information visit https://societyforimplementationresearchcollaboration.org/mechanisms-network-of-expertise/) to convene virtually quarterly to generate and synthesize multiple inputs and then in-person in “deep dives” to articulate a research agenda that would promote the study of implementation mechanisms. The work is being advanced by four working groups organized by (1) causal theory and context, (2) measures and methods, (3) design and analysis, and the (4) linkages between implementation strategies, mechanisms, and outcomes. MNoE members joined the IDWs, convened in workgroups, hosted open breakout sessions for SIRC attendees to contribute to the agenda, served as plenary speakers, and synthesized learnings to close out the conference. SIRC attendees completed evaluations of the MNoE activities and appreciated the highly interactive, facilitated nature of the breakout working meetings, and indicated that they had a much better understanding of implementation mechanisms. The MNoE work continued after the conference with the goal of disseminating the ultimate research agenda at SIRC’s 6th conference.

## Committee membership and abstract review procedures

The program committee comprised 19 participants. Committee members were selected to ensure representation from multiple viewpoints. Members were from four different countries, various career levels, and represented researchers, intermediaries, practitioners, and policy makers.

The abstract review committee comprised 39 participants and, like the program committee, was selected to ensure diverse representation and ensure that submitted abstracts would be reviewed by those with similar roles within implementation science. Each abstract was independently reviewed and scored by two committee members. Scores were averaged across reviewers unless the scores were substantially discrepant, in which case a third reviewer provided additional perspective to determine the abstract score. The abstract review criteria consisted of six components, including alignment with the conference theme, the inclusion of partnerships, background, methods or conceptual fit (if not a research study), findings or results, and implementation focus. Ultimately, the inclusion of partnerships proved difficult to score, so this component was not incorporated in final scores. Raters were given the opportunity to provide additional comments or indicate if the abstract score was not reflective of the overall merit of the proposal. A specified score cutoff was used based upon the number of available breakout rooms. Abstracts with scores at or above this number were provisionally accepted. A separate cutoff was used to determine poster acceptances. Paper presentations were then examined to ensure representation from people of color, women, international teams, and to confirm a balance of research, practice, and policy. Modest modifications to the program were then made. The SIRC 2019 conference received 354 individual submissions, more than doubling the number of submissions received in 2017 for the previous conference and five times more than the inaugural conference in 2011. Seventy-three presentations were accepted as plenaries or breakout symposia. An additional 129 posters were accepted.

Spearheaded by the awards committee, SIRC developed new awards in addition to two standing conference awards (Student, New Investigator). Three new conference awards were developed to acknowledge presentations that highlighted the conference theme of science-practice integration, as well as the work of implementation practice. The SIRC Student Award was presented to Heather L. Bullock, for the presentation she led with colleagues John N. Lavis, Michael G. Wilson, Gillian Mulvale, and Ashleigh Miatello. The SIRC New Investigator Award was presented to Emily Becker-Haimes, for the presentation she led with colleagues Jessica Fishman, Torrey Creed, Courtney Benjamin Wolk, Danielle Centeno, and David Mandell. The SIRC Conference Theme Award for a Symposium was presented to Chair: Briana S. Last, Discussant: Gregory Aarons, and co-authors/presenters: Courtney Benjamin Wolk, Rinad S. Beidas, Andrew Quanbeck, Nathaniel Williams, Rebecca Stewart, David S. Mandell, Heather Nuske, and Emily Becker-Haimes. The SIRC Conference Theme Award for an Oral Presentation was presented to Brittany Cooper, Adam Darnell, Angie Funaiole, Kevin Haggerty, and Laura Hill. The SIRC Implementation Practice Award was presented to Robert Franks and Jonathan Scaccia. In addition to the conference awards, we announced the inaugural SIRC Mission Award to acknowledge a team that has made outstanding collaborative contributions to implementation science and practice. The 2019 awardee was Penn Center for Mental Health & Philadelphia Department of Behavioral Health for their work implementing evidence-based practices in Philadelphia behavioral health care systems.

## Journal update

SIRC began conceiving of a new journal at the 2015 conference when we held an open session to discuss the need, scope, and path for such an endeavor. We convened a formal Planning Committee and held an inaugural meeting at our 2017 conference to solidify details and launch monthly meetings for a workgroup led by co-founding editors-in-chief Cara Lewis and Sonja Schoenwald. SIRC’s journal proposal was positively received by several publishers with whom we met on several occasions to ensure a competitive scope for the expanding market. In 2018, we secured seven inaugural Associate Editors including Danny Almirall, Rinad Beidas, David Bradford, Aaron Lyon, Larry Palinkas, Michael Southam-Gerow, and Terje Ogden. In 2019, after much deliberation and many iterations, we solidified the title and scope of the SIRC journal. *Implementation Research and Practice* is an international, peer-reviewed, open access, online-only journal providing rapid publication of interdisciplinary research that advances the implementation in diverse contexts of effective approaches to assess, prevent, and treat mental health, substance use, or other addictive behaviors, or their co-occurrence, in the general population or among those at-risk or suffering from these disorders (https://us.sagepub.com/en-us/nam/implementation-research-and-practice/journal203691). At the time of the conference, we had not yet announced the publisher. At the conference, the editors-in-chief described the submission process and invited manuscripts for consideration in the inaugural year.

## Summary

This supplement offers a compilation of the abstracts of the oral and poster presentations from the 2019 Society for Implementation Research Collaboration (SIRC) Conference, “Where the Rubber Meets the Road: The Intersection of Research, Policy, and Practice.”

## Data Availability

Not applicable.

## Abbreviations

CEO: Chief executive officer
IDW: Implementation development workshop
NIH: National Institutes of Health
NIMH: National Institute of Mental Health
NoE: Network of Expertise
MNoE: Mechanisms Network of Expertise
SIRC: Society for Implementation Research Collaboration
